# Outcomes of Equity-Oriented, Web-Based Parenting Information in Mothers of Low Socioeconomic Status Compared to Other Mothers: Participatory Mixed Methods Study

**DOI:** 10.2196/22440

**Published:** 2020-11-10

**Authors:** Pierre Pluye, Reem El Sherif, Araceli Gonzalez-Reyes, Emmanuelle Turcotte, Tibor Schuster, Gillian Bartlett, Roland M Grad, Vera Granikov, Melanie Barwick, Geneviève Doray, François Lagarde, Christine Loignon

**Affiliations:** 1 Department of Family Medicine McGill University Montreal, QC Canada; 2 School of Information Studies McGill University Montreal, QC Canada; 3 Child Health Evaluative Sciences Research Institute, The Hospital for Sick Children University of Toronto Toronto, ON Canada; 4 Lucie and André Chagnon Foundation Montreal, QC Canada; 5 Department of Family Medicine Université de Sherbrooke Sherbrooke, QC Canada

**Keywords:** consumer health information, child development, child health, literacy, information outcomes

## Abstract

**Background:**

Typically, web-based consumer health information is considered more beneficial for people with high levels of education and income. No evidence shows that equity-oriented information offers equal benefits to all. This is important for parents of low socioeconomic status (SES; low levels of education and income and usually a low level of literacy).

**Objective:**

This study is based on a conceptual framework of information outcomes. In light of this, it aims to compare the perception of the outcomes of web-based parenting information in low-SES mothers with that of other mothers and explore the perspective of low-SES mothers on contextual factors and information needs and behavior associated with these outcomes.

**Methods:**

A participatory mixed methods research was conducted in partnership with academic researchers and Naître et grandir (N&G) editors. N&G is a magazine, website, and newsletter that offers trustworthy parenting information on child development, education, health, and well-being in a format that is easy to read, listen, or watch. *Quantitative component (QUAN)* included a *3-year longitudinal observational web survey*; participants were mothers of 0- to 8-year-old children. For each N&G newsletter, the participants’ perception regarding the outcomes of specific N&G webpages was gathered using a content-validated Information Assessment Method (IAM) questionnaire. Differences between participants of low SES versus others were estimated. *Qualitative component (QUAL) was interpretive*; participants were low-SES mothers. The thematic analysis of interview transcripts identified participants’ characteristics and different sources of information depending on information needs. Findings from the two components were integrated (*QUAN+QUAL integration*) through the conceptual framework and assimilated into the description of an ideal-typical mother of low SES (Kate). A narrative describes Kate’s perception of the outcomes of web-based parenting information and her perspective on contextual factors, information needs, and behavior associated with these outcomes.

**Results:**

QUAN—a total of 1889 participants completed 2447 IAM responses (50 from mothers of low SES and 2397 from other mothers). N&G information was more likely to help low-SES participants to better understand something, decrease worries, and increase self-confidence in decision making. QUAL—the 40 participants (21 N&G users and 19 nonusers) used 4 information sources in an iterative manner: websites, forums, relatives, and professionals. The integration of QUAN and QUAL findings provides a short narrative, Kate, which summarizes the main findings.

**Conclusions:**

This is the first study comparing perceptions of information outcomes in low-SES mothers with those of other mothers. Findings suggest that equity-oriented, web-based parenting information can offer equal benefits to all, including low-SES mothers. The short narrative, *Kate*, can be quickly read by decision policy makers, for example, web editors, and might encourage them to reach the underserved and provide and assess trustworthy web-based consumer health information in a format that is easy to read, listen, or watch.

## Introduction

### Problem and Objectives

This paper explores the outcomes of equity-oriented, web-based, consumer health information from the perspective of young children’s mothers who have a low socioeconomic status (SES). Equity-oriented interventions attempt to move toward equity, that is, reduce inequalities (systematic differences among social groups), though full equity may never be achieved [1]. Education and income are among the most important indicators of SES and are strongly associated with individual and population health status [2-4]. With respect to web-based consumer health information, the literature suggests the following stereotypical inequality: although information in general can help people of low SES, it mainly offers benefits to people of higher SES, that is, those who are the most educated and wealthiest. It is unclear whether equity-oriented information, that is, trustworthy information provided in a format that is easy to read, listen, or watch, can offer equal benefits to all, including low-SES mothers.

Regarding general outcomes of web-based information at the population level, the literature suggests mixed positive and negative outcomes. First, health services research suggests that browsing the internet leads to a decrease in unnecessary calls and visits to health professionals and helps to optimize services utilization [5,6]. Specifically, consumers’ use of trustworthy sources of web-based health information is associated with improved knowledge, empowerment, self-care, engagement in health care, health outcomes, and quality of life [6-12]. Second, exposure to web-based information can lead to negative outcomes such as worries and anxiety, for example, cyberchondria, deterioration of the patient-provider relationship, and unnecessary visits to the emergency room [13-18].

However, 4 literature reviews have shown that specific outcomes of information are rarely researched [19-22]. Typically, information outcomes are diluted in the outcomes of educational programs and communication with professionals [23-25]. With respect to the health of the mother and child, studies have examined the outcomes of lay pediatric information in the context of the effectiveness of parent-child professional communication and patient education programs. The studies concluded that the parents’ level of health literacy is positively associated with the quality of pediatric care, compliance with medical interventions, and child health outcomes but negatively associated with medication errors and visits to emergency rooms [26,27]. This led to the inclusion of the *Low Health Literacy Universal Precaution Principle* in medical education and continuing professional development programs [28-30], which recommends the universal provision of clear lay information in all patient-clinician encounters.

Such principles are essential for web-based consumer health information because the proportion of people with a low level of literacy is substantial; for example, more than 50% of Canadian adults have a low literacy level [31]. People with a low literacy level can read, listen to, watch, and understand one-idea sentences in plain language, but they arguably face difficulties in finding trustworthy web-based information, critically appraising it, and understanding nuanced ideas or specialized language [31]. It follows, therefore, that their web-based health information literacy level is also low, which comprises computer literacy, information literacy, and health literacy. This is particularly important for parents because their low literacy level is detrimental to child health education, healthy behaviors, health, and medication [32-34]. Parents with low education and low income, hereafter referred to as low-SES parents, typically have a low literacy level, yet they have greater information needs compared with their higher-SES counterparts [20].

In the information sciences, we found 5 studies that focused on parenting information needs and information-seeking behavior and included low-SES parents. Two surveys, one in Australia and one in Switzerland, found no statistical difference in internet utilization between low-SES parents and other parents [35,36]. Two qualitative studies on mothers’ information needs (mostly middle-class mothers) suggest that practical information on mothering produced by professionals is valuable and helpful [37,38]. One study found that all low-SES mothers search for health information on the internet [39].

In addition, little research has specifically focused on outcomes of web-based parenting information reported by low-SES mothers [40,41]. We found little evidence (and some of it contradictory) on parental perception of outcomes of web-based parenting information, namely information on child education, development, health, and well-being. One study suggested that all parents reported a similar degree of satisfaction with web-based parenting information in general [42]. Five studies suggested that trustworthy web-based information, that is easy to read, listen to, or watch, can improve the quality of life of parents of low SES (including refugee and homeless) and have positive family, economic, and social impacts [43-47]. In contrast, 4 studies showed that barriers persist regarding the acquisition, cognition, and application of web-based parenting information, which are associated with information-related inequalities, perpetuating the digital divide between low-SES and high-SES parents [40,48-50].

However, no study has compared the low-SES parents’ perception of outcomes of equity-oriented web-based information with that of other parents. In light of this, this study aims to (1) compare the low-SES mothers’ perception of outcomes of web-based parenting information with that of other mothers, and (2) explore the perspective of low-SES mothers on contextual factors and information needs and behavior associated with these outcomes.

### Background

This study is based on a partnership between Naître et grandir (N&G) and McGill University (Information Assessment Method, IAM). N&G is funded by the Chagnon Foundation, a philanthropic organization that seeks to prevent poverty. N&G produces a magazine and a website with an email newsletter (in French) for parents of 0- to 8-year-old children.

N&G seeks to raise societal awareness of the importance of early child development for enabling conditions of educational success. It provides free, independent, and trustworthy parenting information to valorize, inform, educate, and equip parents and families of young children, especially among vulnerable populations, for example, tips for parents, dos and don’ts validated by experts. Specifically, N&G provides web-based parenting information content that is easy to read, listen to, and watch, specifically webpages with corresponding videos, podcasts, and computer-audio-assistant highlighting sentences read. The website content is organized by age groups and topics.

N&G is widely read by French-speaking parents across Canada, the United States, and more than 100 other countries. In the 2018 calendar year, 61.6 million N&G webpages were viewed during 35.3 million visits to the N&G website by 15.2 million unique internet protocol (IP) addresses across the world. Among those, 20.2 million webpages were viewed during 11.2 million visits to the N&G website by 3.3 million unique IP addresses in Quebec. More than 213,000 N&G weekly newsletters were emailed to parents in Quebec. *SOM Recherche & Sondages* (personal communication, 2015) conducted a survey of a representative random sample of the population of parents of young children in Quebec, which showed that 82% of respondents knew N&G and that 76% consulted it.

### Conceptual Framework

#### Origin

**Figure 1:**
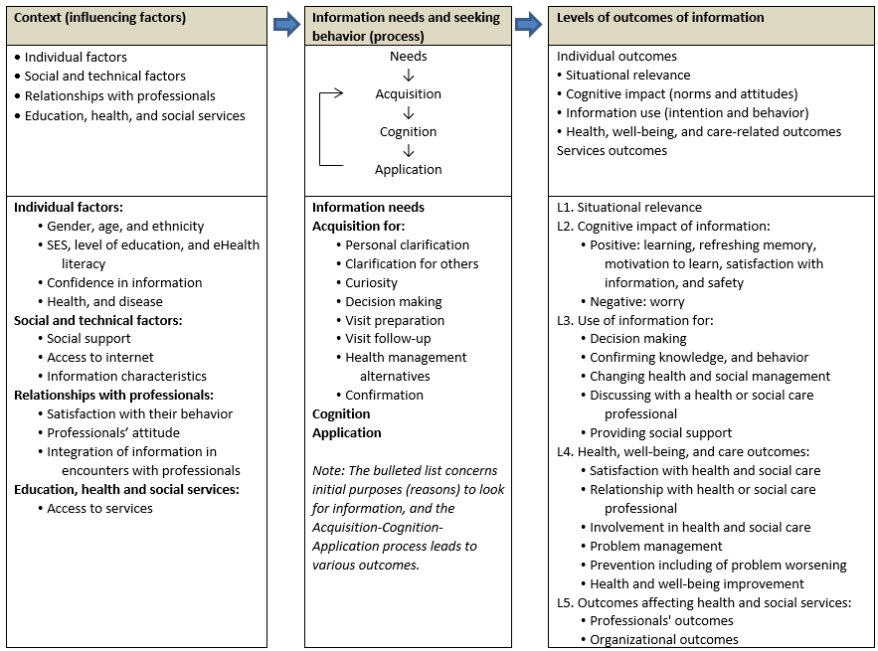
Outcomes of web-based parenting information: conceptual framework. SES: socioeconomic status.

Our conceptual framework is summarized in Figure 1 and published elsewhere [51]. The framework is based on a mixed studies systematic review with framework synthesis of qualitative and quantitative evidence on the outcomes of web-based consumer health information in community-based primary care. In accordance with Gregor’s definition of theories [52], our framework seeks to explain what these outcomes are (five levels of information outcomes), why and how they occur (information needs and seeking behavior), and when, where, and for whom they occur (contextual influencing factors).

#### Five Levels of Information Outcomes

The framework includes 4 individual levels of information outcomes (situational relevance, cognitive impact, use, and health and well-being outcomes of information) and one organizational-level outcome (information outcomes affecting educational, health, and social care services).

- Level 1—situational relevance: Relevance of information is the first value of information [53], and situational relevance is the first outcome of information for a person in a specific situation. Parents read or listen to a webpage when information is relevant to their needs, and they skip it when it seems irrelevant.
- Level 2—cognitive impact: Relevant information content has a cognitive impact that parents may perceive as either positive or negative. For example, they can learn either something new from the information they read, hear, or watch, or they may not understand this information. Relevant information with a positive cognitive impact is usually (but not necessarily) used.
- Level 3—information use: Parents can use information content in 4 different ways: conceptual, legitimating, symbolic, and instrumental. For instance, they may use it to decide to consult health professionals (instrumental use) and then share it with a professional (symbolic use). Information use does not necessarily lead to behavior change.
- Level 4—health and well-being outcomes: Information can benefit, or negatively affect, parents’ and children’s health and well-being, for example, parents might feel reassured (positive outcome) or more anxious (negative outcome) from using information.
- Level 5—organizational outcomes: At this level, parents’ information use may influence their utilization of educational, health, and social care services.

#### Context, Information Needs, and Information-Seeking Behavior

In the framework, information outcomes depend on the context, and parents’ information needs and information-seeking behavior. Regarding *context (influencing factors)*, parents’ individual and social factors are interrelated because the latter, for example, social support for finding, understanding, and using relevant information, can overcome the former, for example, low level of literacy. These factors are listed in Figure 1. Regarding *information needs and seeking behavior*, outcomes of web-based parenting information and contextual factors are defined in relation to a specific situation: a singular content is sought, acquired, or delivered, for example, a webpage, in a particular situation to address the parents’ needs. These conditions are necessary to observe the *imbrication* between information content and information technology and parents—the ultimate decision makers about the value of the information content [54]. In our framework, parents’ information-seeking behavior is defined as the iterative imbrication-centered acquisition, cognition, and application of information.

In accordance with the conceptual framework, our specific research questions were as follows:

- Q1: To what extent do mothers of low SES perceive outcomes of N&G web-based parenting information compared with other mothers?
- Q2: What are the experiences of low-SES mothers with web-based parenting information from N&G compared with other sources of information?

Specifically regarding Q2, we wanted to identify the differences between N&G users and nonusers regarding the factors related to information outcomes (contextual factors, and information needs and seeking behavior).

## Methods

### Approach

We followed an organizational participatory research approach that blends action research and organizational learning to engage organization members to improve practice [55,56]. Researchers and N&G editors partnered throughout the study, developing the research questions together, and making other key research decisions jointly regarding the collection and analysis of data and the interpretation and dissemination of results.

### Design

We used a mixed methods convergent design [57,58]. Mixed methods are crucial for *unpacking complexity* in research on poverty and vulnerability [59]. In our study, mixed methods were justified because our quantitative data collected with the McGill IAM questionnaire reflect the importance of the information outcomes perceived by low-SES parents, and qualitative data were required to gain insight into the mechanisms underlying these outcomes. Thus, to respond to our first research question, we conducted a 3-year longitudinal observational quantitative component of the mixed methods study. To answer the second research question, we conducted a qualitative interpretive study on parental characteristics, context, information needs, and information-seeking behavior (the qualitative component of the mixed methods study). Then, we integrated these components via the assimilation of quantitative and qualitative results [58].

### Quantitative Longitudinal Observational Component

In line with the literature on patient-reported outcome measures, that is, measurement from patients’ perspectives rather than from medical or biological ones [60], we focused on parent-reported information outcomes.

#### Setting and Participants

People can sign up to receive a weekly newsletter containing links to three N&G webpages tailored to their needs (pregnancy; child 0-1 year, 2-3 years, 4-5 years, and 6-8 years). Participants were recruited from mothers of children 0 to 8 years old from Quebec, identified through the postal code and child’s age provided when signing up. They were recruited when they completed at least one IAM questionnaire during the 3-year study period (January 1, 2016, to December 31, 2018, inclusively) and reported an intention to use N&G information for themselves or their child. No incentive was provided to participate.

#### Hypotheses

According to the literature on the digital divide, we hypothesized that low-SES mothers of 0- to 8-year-old children (ie, mothers with a low level of income and a low level of education) are less likely to perceive positive outcomes from N&G information compared with mothers of higher SES. According to the Quebec poverty line definition, a low level of income is an annual family income of less than Can $40,000 (US $30,070). A low level of education constitutes a high school diploma (grade 12, no university degree) or no diploma (high school not completed).

#### Instrumentation

The IAM questionnaire was used to assess the information provided on each N&G webpage. The IAM allowed participants to report perceived outcomes associated with information content of a specific webpage in terms of situational relevance, cognitive impact, intention to use, and expected health outcomes [61]. The development of the IAM questionnaire has been based on a theory, literature review, expert panel, and interviews with consumers [62]. N&G editors and McGill researchers have been partnering since 2014 for collecting parents’ IAM ratings, and they use IAM-based feedback comments to improve information content (crowdsourcing developmental evaluation) [63].

For each N&G webpage, participants were invited to complete an IAM-parent-v2015 questionnaire by clicking on a lateral tab *survey*. Then, respondents had the opportunity to provide written open-ended feedback about the N&G webpage. To decrease response fatigue, respondents did not receive another invitation during the 30-day period following their completion of the IAM. In preparation for this study, we conducted an ecological content validation of the IAM questionnaire with parents who used the initial version of IAM to rate N&G information [64]. In other words, we questioned N&G IAM respondents regarding the relevance and representativeness of the IAM questions [65,66]. We measured the relevance of IAM questions using the IAM ratings of 22,407 parents, and qualitatively evaluated question representativeness and clarity via interviews with a purposeful sample of 21 parents who used N&G. On the basis of quantitative and qualitative results, McGill researchers and N&G editors revised and clarified the IAM questions. This led to the creation of content-validated IAM-parent-v2015.

#### Data Source

All participants were asked to complete a demographic questionnaire when they completed their first IAM questionnaire. All IAM and demographic questionnaires were collected by N&G, anonymized (email addresses replaced by an individual identification number), linked, and transferred to a password-protected server for access by the academic members of the research team.

#### Statistical Analysis

Descriptive statistics were calculated using SAS 9.4 software (SAS Institute). Categorical variables were described as counts and percentages. Ninety-five percent confidence intervals for differences in the proportion of IAM ratings were calculated to estimate outcome differences between the 2 primary study groups: participants combining low level of education and low level of income versus other participants [67,68].

### Qualitative Interpretive Component

#### Reporting

This component is reported in accordance with the Consolidated Criteria for Reporting Qualitative Research [69]. Interviews were conducted with a purposeful sample of 40 low-SES mothers of 0- to 8-year-old children geographically dispersed across Quebec: 21 N&G users and 19 nonusers. Interviews and data analyses were conducted in French. Selected excerpts of transcripts were translated in English for submitting this study to the Journal.

#### Participants

Women who satisfied the following criteria were purposively recruited through emailed invitations: mother of at least one child aged between 0 and 8 years with a low level of income and a low level of education as defined in the Hypotheses section. We sought to recruit mothers from single- and dual-parent families and living in urban, semiurban, and rural areas. The recruitment strategy involved sending an invitation to mothers who used N&G and had agreed to be contacted for research purposes. Only 3 participants were recruited in this manner. Thus, we pursued recruitment aided by a survey firm. In total, 45 persons were contacted, but 5 were not selected because they did not satisfy the eligibility criteria.

#### Data Collection

Data collection took place between January and March 2018. Three academic team members (PhD degrees), experts in qualitative research with vulnerable populations (a male anthropologist, a female bioethicist, and a female sociologist with 10-year experience in social work), met participants in either their home or in a research facility room, as per their choice. Individual face-to-face, semistructured interviews of approximately 1-hour duration were conducted. All participants provided formal written consent before the interviews and were compensated for their time following the interview. Participants who were interviewed in their homes received Can $50 (US $38), whereas those who traveled to a research facility received Can $60 (US $45). Interviews were audio-recorded and transcribed verbatim. On the basis of conceptual framework, the research team worked together in an iterative manner to develop the interview guide. Then, the guide was pilot-tested with 2 mothers of young children, a research trainee not working on the project, and a mother of low SES, and revised accordingly. The guide included themes and probes for both N&G users and nonusers. Specifically, all participants were asked about their use of the internet, their information needs, and information-seeking behavior regarding their child’s development, education, health, and well-being. The N&G information user participants were asked additional questions about their experience with N&G.

#### Data Analysis

**Figure 2:**
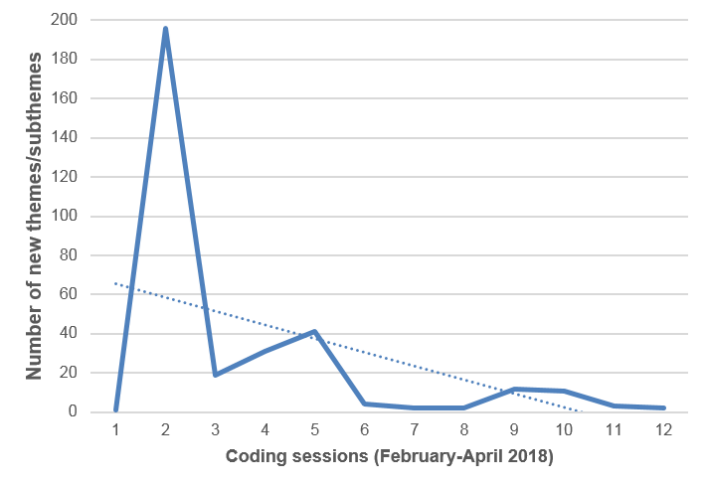
Qualitative data analysis: saturation of themes reached after 10 coding sessions.

Data analysis was performed between February 2018 and May 2019. Transcripts were analyzed using NVivo version 11 (NVivo Qualitative Data Analysis Software, QSR International), which helped to maintain a transparent relationship between data (excerpts of transcripts) and themes and subthemes (auditable trail). Two research professionals (interviewers) read transcripts several times to become fully acquainted with the content before coding the data and used hybrid deductive-inductive thematic analysis [70,71]. An example of the analytical process is presented in Multimedia Appendix 1. They co-constructed a theme tree codebook, using the conceptual framework in a deductive manner, and as new themes and subthemes were found in the data, they were added to this theme tree in an inductive manner. The codebook is presented in Multimedia Appendix 2. All 40 transcripts were analyzed. As shown in Figure 2, saturation of themes and subthemes was reached after 10 coding sessions, that is, once 21 of the 40 interview transcripts had been analyzed. Only 1.5% (5/325) of new themes and subthemes were found in the remaining 19 interviews.

With respect to methodological rigor, data analysis involved multiple sessions where themes, subthemes, and the assignments of transcript excerpts to themes and subthemes were discussed (corroborated or nuanced) among researchers. The reliability of the 2 research professionals conducting the thematic analysis was ensured via the co-construction of the theme tree, initial paired coding sessions on few transcripts, memo writing, and regular meetings with the principal investigators and then with coinvestigators and N&G editors to discuss analysis issues and new themes [70].

### Integration of Quantitative and Qualitative Components

The quantitative and qualitative components were integrated in two ways. First, they were integrated using the conceptual framework [57]. Quantitative observations provided results on low-SES mothers’ reported outcomes compared with other mothers. Qualitative observations produced complementary results on low-SES mothers’ information outcome–related contextual factors, information needs, and information-seeking behavior.

Second, quantitative and qualitative results were assimilated as follows [58]: the principal investigator transformed quantitative results into a narrative, which was combined with an interpretation of qualitative results for building a comprehensive ideal type of low-SES maternal perception of outcomes of web-based parenting information, including outcomes-related factors. All coauthors reviewed the ideal type. According to Weber [72], better understanding can emerge from the scientific construction of an ideal type; here, *ideal* refers to ideas, not to an ideal to achieve. An ideal type does not exist *in the real world under this pure, absolutely ideal form* [72]. It integrates common aspects of a phenomenon in a comprehensive and coherent manner. This integration adds value to qualitative and quantitative results alone.

## Results

### Quantitative Results

Over the 3-year study period, 1889 participants completed 2447 IAM questionnaires (on average 1.3 questionnaires per participant; range 1-12) pertaining to 683 distinct N&G webpages (on average 3.6 questionnaires per page; range 1-26). Participants were geographically dispersed across 352 of the 420 Quebec postal codes (352/420, 83.8%)*.* Participants’ demographic information, IAM ratings, and comments are detailed below.

Among participants 89.31% (1687/1889) were aged between 25 and 44 years, 94.12% (1778/1889) were living with a partner, 85.23% (1610/1889) were living full time with their child; 11.54% (218/1889) had an annual family income less than Can $40,000 (US $30,070), and 4.71% (89/1889) did not complete high school or had only a high school diploma (grade 12). Compared with the 25- to 64-year-old Quebec population, participants had a higher level of education (Table 1). Compared with couples with children in Quebec, participants had a lower level of income (Table 2). Among participants, 77.77% (1469/1889) provided a valid postal code, and according to the Material and Social Deprivation Index [73], 70.30% (1328/1889) of participants had low levels of education and income and 35.2% (665/1889) of other participants lived in deprived areas.

**Table 1:** Level of education: participants of the quantitative component versus Quebec 25- to 44-year-old population.

Population	Diploma, n (%)			
	None	Secondary school (grade 11 or less)	College (grade 12 or 13)	University
Participants (n=1889)	21 (1.11)	210 (11.12)	430 (22.76)	1228 (65.01)
Quebec^a^ (n=2,056,110)	215,891 (10.50)	709,358 (34.50)	483,186 (23.50)	647,675 (31.50)

^a^Source: Statistics Canada Census of population: Table 37-10-0099-03 distribution of the population of 25 to 64 years old by the highest level of education. Ottawa: Government of Canada; 2016.

**Table 2:** Level of income: participants of the quantitative component versus couples with children in Quebec.

Population (N; %)	Annual family income, Can $ (US $), n (%)		
	<40,000 (30,070)	40,000-80,000 (30,070-60,140)	80,000 (60,140)
Participants (n=1889)	217 (11.49)	586 (31.02)	1086 (57.49)
Quebec^a^ (n=911,975)	49,835 (5.47)	319,170 (35.00)	542,970 (59.54)

^a^Source: Statistics Canada Census of population: Catalog no. 98-400-x2016129. Ottawa: Government of Canada; 2016.

The types of information outcomes are detailed in Table 3. Specifically, all ratings (2447/2447, 100.00%) reported the participants’ intention to use N&G information for themselves or for their child, at least in a conceptual manner. In 97.79% (2393/2447) of ratings, participants expected that information use would lead to health and well-being benefits for themselves or their child.

**Figure 3:**
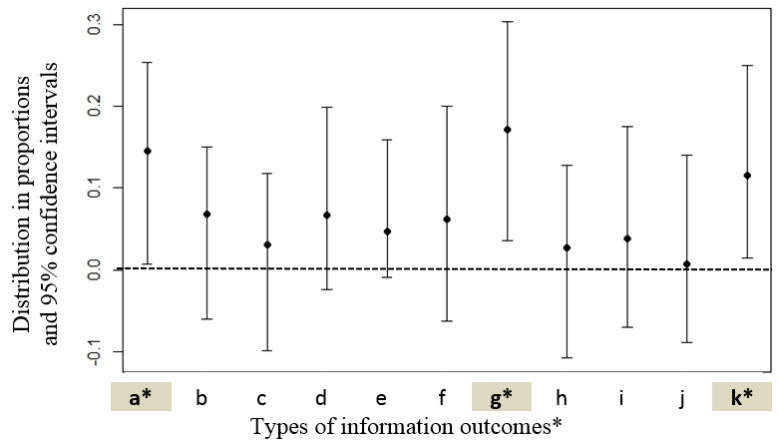
Types of information outcome: differences in proportions comparing the Information Assessment Method ratings of participants combining low education and low income versus other participants.

Figure 3 displays the estimated group differences in proportions for these outcomes, along with 95% CI. The comparison of the different types of intention to use and expected health and well-being benefits of N&G information between the IAM ratings of the participants with low levels of education and income (n=50) and those of the other participants (n=2397), suggested that 5 of 11 outcomes were not inferior for the former group (items a, d, e, g, and k in Figure 3), including 3 being superior (items a, g, and k in Figure 3). The results do not provide conclusive evidence for differences (or the absence of such differences) regarding the 6 other types of outcomes (items b, c, f, h, i, and j in Figure 3). In other words, N&G information was more likely to help participants with low levels of education and income to better understand something (mean difference 0.144; 95% CI 0.007-0.253), decrease worries (mean difference 0.171; 95% CI 0.036-0.302), and increase self-confidence in making a decision with someone else (mean difference 0.115; 95% CI 0.015-0.249).

**Table 3:** Quantitative component—perceived information outcomes: Information Assessment Method ratings of participants with a low level of education and income versus other participants.

IAM^a^ questions and response options		Low-education and low-income mothers (50 IAM ratings), n (%)	Other mothers (2397 IAM ratings), n (%)	All participants (2447 IAM ratings), n (%)
**Q1. Is this information relevant?**				
	Very relevant (this is the information I expected)	32 (64)	1629 (67.96)	1661 (67.88)
	Relevant	17 (34)	748 (31.21)	765 (31.26)
	Somewhat relevant	0 (0)	17 (0.71)	17 (0.69)
	Very little relevant (this is not the information I expected)	1 (2)	3 (0.13)	4 (0.16)
**Q2. Did you understand this information?**				
	Very well (I understood everything)	45(90)	2243 (93.58)	2288 (93.50)
	Well	5 (10)	152 (6.34)	157 (6.42)
	Poorly	0 (0)	1 (0.04)	1 (0.04)
	Very poorly (I did not understand much)	0 (0)	1 (0.04)	1 (0.04)
**Q3. What do you think about this information? Check all that apply.**				
	This information allowed me to validate what I do or did	30 (60)	1655 (69.04)	1685 (68.86)
	This information taught me something new	22 (44)	1207 (50.35)	1229 (50.22)
	This information reassured me	23 (46)	995 (41.51)	1018 (41.60)
	This information refreshed my memory	12 (24)	787 (32.83)	799 (32.65)
	This information motivated me to learn more	17 (34)	472 (19.69)	489 (19.98)
	There is a problem with this information	1 (2)	28 (1.17)	29 (1.19)
	I disagree with this information	0 (0)	13 (0.54)	13 (0.53)
	This information can have negative consequences	0 (0)	5 (0.21)	5 (0.20)
**Q4. Will you use this information?**				
	Yes	50 (100)	2397 (100.00)	2447 (100.00)
	No	0 (0)	0 (0)	0 (0)
**Q4a. How you will use this information for you or your child? Check all that apply.**				
	This information will help me to better understand.	36 (72)	1345 (56.11)	1381 (56.44)
	I will use this information to do something in a different manner.	9 (18)	584 (24.36)	593 (24.23)
	I will use this information to discuss with someone else.	9 (18)	543 (22.65)	552 (22.56)
	I did not know what to do, and this information will help me to do something.	11 (22)	356 (14.85)	367 (15.00)
	I knew what to do, and this information convinced me to do it.	5 (10)	123 (5.13)	128 (5.23)
**Q5. Do you expect any benefit for you and your child from using this information? Check all that apply.**				
	This information will help me to improve the health or well-being of my child.	31 (62)	1603 (66.88)	1634 (66.78)
	This information will help me to be less worried.	27 (54)	858 (35.79)	885 (36.17)
	This information will help me to prevent a problem or the worsening of a problem.	12 (24)	676 (28.20)	688 (28.12)
	This information will help me to handle a problem.	14 (28)	615 (25.66)	629 (25.70)
	I will be better prepared to discuss with someone else.	11 (22)	547 (22.82)	558 (22.80)
	I will be more confident to decide something with someone else.	13 (26)	335 (13.98)	348 (14.22)
	I expect no benefits.	1 (2)	53 (2.21)	54 (2.21)

^a^IAM: Information Assessment Method.

### Qualitative Results

#### Participants’ Characteristics

The participant characteristics are described in Table 4. Forty low-SES mothers with at least one child aged between 0 and 8 years were interviewed: 21 N&G information users and 19 nonusers. On average, they had 2 children (range, 1–5 years; median, 2), including at least one child aged 0 to 8 years (mean 5.9 years, range pregnancy to 8 years, and median 4 years). Interviews revealed that about half were just above the poverty line (eg, Can $40,000-50,000 [US $30,070-38,085] per year) or the low level of education (eg, professional certificate).

The results reveal no major differences between N&G information users and nonusers with respect to the frequency of internet use and the type of information retrieved. Participants mentioned child education, entertainment, goods, health, news, recipes, social media, and transportation regardless of whether (1) they used N&G information or not; (2) where and when the internet is used at home, public, or work setting, anytime but mostly in the evening; and (3) the languages in which the internet is used, that is, mostly French, English, and Spanish.

**Table 4:** Qualitative component: sociodemographic characteristics of the participants (interviewees).

Characteristics		Participants, n (%)
**Utilization of N&G^a^**		
	Users	21 (53)
	Nonusers	19 (48)
**Language spoken at home**		
	French	27 (68)
	French and English	3 (8)
	French and Spanish	2 (5)
	Spanish	3 (8)
	French and Romanian	1 (3)
	Unknown	4 (10)
**Education**		
	General education (grade 11 or less)	16 (40)
	Professional education (grade 9, 10 or 11)	8 (20)
	College (grade 12 and 13 in-progress or completed)	16 (40)
	University	0 (0)
**Annual family income, Can $ (US $)**		
	Less than 10,000 (7917)	0 (0)
	10,000-20,000 (7917-15,234)	5 (13)
	20,000-30,000 (15,234-22,851)	14 (35)
	30,000-40,000 (22,851-30,070)	19 (48)
	40,000-50,000 (30,070-38,085)	2 (5)
	Over 50,000 (38,085)	0 (0)
**Parental status**		
	Single mother	16 (40)
	Couple	23 (58)
	Not specified	1 (3)
**Housing**		
	Rent	29 (73)
	Own	7 (18)
	Not specified	4 (10)

^a^N&G: Naître et grandir.

#### Participants’ Informational Context

The contextual factors were as follows. Regarding individual characteristics, on average, participants were 32 years old; 24 were seeking a preuniversity degree equivalent to 2 years of general or professional education after grade 11, and the other 16 had stopped their education after grade 11. Interviews analysis revealed that 16 participants were single mothers who earned less than Can $25,000 (US $19,043) per year, 22 other participants had an annual family income of less than Can $40,000 (US $30,070), and 2 participants earned between Can $40,000 and Can $50,000 (between US $30,070 and US $38,085). Furthermore, 18 participants mentioned that one of their children had a health or developmental problem (12/21 N&G information users vs 6/19 nonusers): attention deficit hyperactivity disorder (n=6), language delay (n=6), cardiac problem (n=2), autism spectrum disorder (n=2), and growth delay (n=2).

As illustrated below, all participants stated that they used the internet several times per day with their cellular phone. Almost all participants considered it easy to access the Web (17/21 N&G information users vs 19/19 nonusers), given, for example, the presence of free Wi-Fi at work, and in numerous public places (shops, schools, etc). All participants reported using their cellular phone to access the internet, whereas only 5 participants used computers or tablets. Participants said they used their cellular phone more frequently because it is more portable and personal, and because it is accessible at all times, unlike a tablet or computer or that can be shared with other family members and, in the case of computers, needs to be booted up. Only 5 participants mentioned barriers to internet access, namely the cost of cell phone data. Two mentioned they had to reduce their data consumption as it was too expensive, and thus accessed the internet using free Wi-Fi when possible:

As soon as you ask yourself a question, you just have to go on the Internet, and you'll get your answer. Now, it seems like we're not used to not knowing something. [...] Right now, you take out your phone, you know it right away [laughs].

D01

Even before, I didn't pay to have it [Internet] on my phone, I just had it at home or in places with Wi Fi; it was pretty much just at home that I used it, but now I have it everywhere I want, well... We can share our connection.

D12

#### Participants’ Information Needs and Seeking Behavior

##### Information Sources

We found few differences between N&G information users and nonusers regarding information needs specific to child development (19/21 N&G users vs 9/19 nonusers) and education (7/21 N&G users vs 1/19 nonusers). Participants identified 4 sources accessed for information seeking: websites, web-based forums, relatives, and care services (no particular difference between users and nonusers). These sources are summarized in Table 5. Depending on information needs, participants described searching multiple sources of information in an iterative manner (including magazines and books from the library, radio, and television).

**Table 5:** Qualitative component: participants’ information-seeking behavior.

Descriptor	Parenting information-seeking behavior			
Sources^a^	Websites: experts providing trustworthy information on websites (eg, N&G^b^)	Web-based forums: credible people with life experience similar to the participant’s experience	Relatives: trusted relatives with children (eg, mothers, sisters, and friends)	Care services: trustworthy professionals from health and social care services^c^
Why	Nonurgent problem Easy (24/7) Autonomy	Nonurgent problem Similar values Breaking isolation	Nonurgent problem Affective support Mutual understanding	Urgent or severe issue High risk Uncertainty
Facilitators	Easy-to-read, listen to, and watch information Corroboration by different websites	Comparison of information content with other sources	Routine information exchanges Knowledge of children of participants	Free easy access to health and social care services Reassurance
Barriers	No lay information Lack of content on child development and education (compared with health)	Lack of quality control Incivilities Lack of content on child development and education	Different child-related values and preferences (eg, generation gap)	Difficulties to navigate services, specifically services addressing behavioral and social issues

^a^The 40 participants address their information needs via one or more of the following sources.

^b^N&G: Naître et grandir.

^c^Including professionals and staff from community organizations.

##### Source 1–Websites

Typically, participants referred to this as *Googling*. Websites without social interaction, such as N&G, were the most common type of first-contact source, especially when seeking information in a nonemergency situation. Governmental and institutional websites were mentioned as sources of trustworthy information. Websites were considered easy to navigate and convenient (available 24/7). Participants reported that they provide a variety of information content on numerous topics and languages, which allows them a degree of autonomy and independence. However, they mentioned that information on child development and education is more difficult to retrieve than general health information, whereas this is not the case in N&G:

What I often do, since I speak all three languages, Spanish, French, English, I go and search [online] in all three languages. So, we often go and see different perspectives of the subject, and then I make my mind up about it. […] That way I have a more global idea.

D10

For me it’s easy because I know how to navigate, I know how to search for information [online]; [...] For example, N&G tab “Age 5-6”: I go there. It’s all there. It’s fast. When you read, for example “at this age, they do role-playing games,” you don't know what role-playing games are, […] you click on it and it takes you to another page that explains what role-playing games are. [...] I find it very practical.

E91

Sure, if it’s anything medical, I know where to go. If it’s dental, I know where to go. But like I think, maybe on a more behavioral level, then I’m like [not knowing where to go].

E01

##### Source 2–Web-Based Forums

Participants considered web-based forums an interesting source for information on general issues, as they provide information from people who have experienced a similar issue, and thus considered credible. Participants sought out these forums for isolation-breaking interactions, specifically looking for people with similar values with whom to interact. They compared the informational content from forums with other sources of information when questioning information quality, for example, the presence of offensive language and inappropriate comments in a forum. As with websites, participants reported that web-based forums lack content on child development and education:

I'm pretty isolated here. My husband, his family is here, so that's why we came here, but otherwise, […] I have no social circle and no family here. So [online forums] make me socialize a little bit with women that I might have some affinity with.

E09

There are 800 of us in that group [online], so there are 800 people who can give me an answer. What’s also good is that there are all kinds of specialties, pharmacists, nurses, physiotherapists, chiropractors... Depending on the question, there’s definitely someone specialized who can answer me in addition to sharing their experience as a mom.

D12

I’m on a mom forum on Facebook, it’s like a private group. So the first thing I usually do is I ask for advice, and that’s for those who have children of the same age. Then I do a little research [online] to compare.

E12

##### Source 3–Relatives

Participants reported that parents and relatives with children constituted a trusted source of information on child development, education, and nonemergency situations. These individuals provided effective support and shared familiar experiences. Mutual understanding between relatives and participants appears to be enabled by a history of information exchanges and relatives’ knowledge of the participants’ children. Not all participants had relatives with children, and some reported different values and preferences compared with their family members, which limited this source:

My first instinct is always to consult someone close to me. It really depends on the moment. If there is no rush and if the person is available, I call on my relatives.

D18

I consult friends who have children, and lastly my mother [laughs].

D19

I often call my mother to find out. But it’s not always good what parents tell you. They often share old wives’ tales, and in their day it worked like that. But now the products have changed and often what my mother tells me is not good.

T05

##### Source 4–Care Services

All participants trusted information provided by professionals working in health and social care services. For example, they never hesitated to call the Quebec HealthInfo 24/7 free phone line or to visit a clinical setting. They used care services when faced with an emergency, such as an accident, perceived potential risks associated with self-care, and uncertainty, for example, hesitant to make a self-diagnosis or to use a homemade remedy. Participants praised easy access to public services and community organizations, and reassurance (decrease worries) obtained from professionals. In turn, this appears to enable and reinforce the use of this source. However, almost all participants reported difficulties in finding information about, and navigating, social care services:

You can ask questions [to pharmacists], and you won’t have to wait seven or eight hours in the emergency room.

D17

My young child has a stye right now. I know for a fact that it is a stye, but I didn’t take a chance and I went to the doctor for the doctor to tell me “yes, that’s what he has, then yes, you’re doing the right thing, keep doing the compresses and that’s it.” I knew what I had to do. I’ve been through this before because my [other] child is 17 and had a stye, so I know what it is, but I need to be reassured.

E03

If [my question] is a medical question, I know where to go. […] At the behavioral level, let’s say I always tend to think: Okay, but who am I going to see?

E01

##### Searching for Multiple Sources of Information in an Iterative Manner

Typically, the participants’ information-seeking behavior consists of an iterative process that includes multiple sources of information. For instance, some participants reported beginning by looking for information on websites because it is convenient. They then consult web-based forums and relatives to search for complementary and corroborating information. Finally, they consult with health and social care services to confirm what they have found or to reduce uncertainty when they find contradictory content from websites, forums, and relatives. Some participants reported that they started their information search by consulting relatives and then sought to corroborate and confirm information obtained using websites, web-based forums, and care services. Participants mentioned that they consult care services eg, HealthInfo 24/7 phone line, directly when their information needs pertain to a perceived emergency. They may then seek corroboration and additional information through websites, forums, and relatives:

I find it easier today with the internet. You have a lot of accessibility. When I have a question, I’ll turn to my sisters, my mom, but if they’re not available, sometimes I’ll just Google my question, and I’ll find forums.

T07

The first reflex is always to consult someone close to you. That depends on the moment. If the question is urgent and we don’t have [access] to the person, we take what we have at hand, the Internet.

D18

When they have a fever, or are sick, or are in pain somewhere, then I’ll go and look at all kinds of places. [...] Calling at places sometimes you can’t get through right away.

D15

### Integration of Quantitative and Qualitative Results: The “Kate” Ideal Type

The ideal type, Kate, is a 30-year-old single mother with 2 children who are 2 and 6 years old. She has completed secondary school and is interested in acquiring professional education. She accesses the internet multiple times daily using her cellular phone. Her annual income is Can $20,000 (US $15,234), and thus she cannot afford additional internet fees to use on a tablet or computer. However, she has easy access to the internet when needed by using her neighbors’ and relatives’ Wi-Fi connection, with their permission, and by using free Wi-Fi services in the mall, a local community organization, and the public library. She says, “I take my phone everywhere and can be on the internet almost any time. It’s mine and is my link to friends, parents, and the world.” When she wants to know something about anything, her first reflex is to look at her phone.

When she needs information regarding the education, health, and well-being of her children, she starts by browsing the top-listed websites on an internet search engine. She compares webpages on a topic from multiple websites, seeking coherence and corroborating information, and confirms her findings using trustworthy governmental and noncommercial websites such as N&G. Usually, she finds relevant and understandable information on N&G that supports what she is planning to do or teaches her something new. Using N&G informational content for her children often decreases her worries and leads her to expect improvement regarding her children’s health or well-being and in her interaction with health or social care professionals. Specifically, she finds that N&G provides valuable information about child behavior, development, and education.

In addition, she seeks complementary information to her *Mom-like-me* social media group, specifically by a group member tagged as a mother and a nurse. Concomitantly, she calls her mother and her best friend, who has children of the same age as hers. In this way, she receives valuable reassurance and emotional support from relatives who know her and her children well. Finally, she considers all of the information she has obtained and filters it through her values, preferences, and financial capabilities to make child-related decisions. She calls the HealthInfo 24/7 phone line or consults with health professionals in the case of a perceived emergency, when she has doubts regarding the information she has obtained, or when she faces contradictory information and uncertainty.

## Discussion

### Principal Findings

Results suggest that in families of young children, there are few differences between low-SES mothers compared with higher-SES mothers with respect to the perceived benefits of equity-oriented, that is, trustworthy and easy-to-read, listen to, and watch N&G web-based parenting information (situational relevance, cognitive impact, use, and subsequent health and well-being outcomes of information). According to our qualitative results, this hypothesis is limited to nonurgent problems because participants primarily turn to professionals for emergency and risk-related information needs. This hypothesis can be tested in future research with other websites using the web version of IAM, or the mobile version for smartphones released in January 2019.

Specifically, these results do not support our literature-based initial hypothesis that participants’ level of education and income would be associated with information benefits. Our results suggest the following hypotheses for future research. Compared with other mothers, low-SES mothers might be more likely to report that web-based parenting information that is trustworthy and easy to read, listen to, and watch helps them to better understand child-related issues and to be less worried and more confident in making decisions with someone else, for example, a professional. They may also report that web-based information helps them to discuss issues with someone else, for example, a relative, and do things differently, better manage or prevent a child behavior problem, improve their health or the health of their child, and be satisfied with professional services.

The results from this evaluation of specific N&G information webpages advance the knowledge on information outcomes. Although mixed methods in research on poverty are expanding rapidly [59], our mixed methods study is the first to compare the outcomes of web-based parenting information from the perspective of low-SES mothers seeking specific child-related information versus other mothers. In this study, the advantages of mixed methods are illustrated by the synergy between our quantitative results on how often information outcomes are reported by low-SES mothers compared with mothers of higher SES and our qualitative results on why and how low SES mothers retrieve and corroborate information.

The low-SES mothers we interviewed frequently access the internet. Moreover, the results of a recent survey of a representative sample of the New York State population (n=1350) suggested that the level of health literacy is not associated with the volume of internet utilization [74]. The low-SES mothers we interviewed considered websites and web-based forums as information sources that are helpful in everyday life. Some relied on their social network, for example, relatives, to obtain information and emotional support before seeking information on websites and web-based forums and from care services. This is congruent with the literature [75-79].

Furthermore, our participants might have been atypical for two reasons. First, they volunteered and, thus, may be biased in support of N&G. Second, although McCloud et al [48] suggested that people of low SES in the United States may experience difficulties accessing the internet despite technical support (provision of computers, home internet connection, and technician help), all of our participants reported that they were able to access the internet easily and none reported technical barriers.

Our study has three main limitations. First, for the quantitative component, we used a convenience sampling strategy (self-selected volunteer participants), and participants did not complete an IAM questionnaire each time they visited the N&G website. This limitation probably led to an overestimation of positive outcomes (social desirability bias). However, assuming this bias influenced all participants in the same manner, regardless of their levels of education and income, this limitation did not affect the statistical analysis. Second, for the qualitative component, about half of the participants were just above the poverty line or the low level of education threshold, which certainly influenced the results. Third, parental efficacy was not examined in our interviews, but this might have affected our data, for example, information-seeking behavior. Numerous studies have shown that poverty has a detrimental effect on parenting, including perceived parental efficacy [80-82]. The concept of perceived parental efficacy is defined as “beliefs or judgments a parent holds on their capabilities to organize and execute a set of tasks related to parenting a child” [83].

In contrast to general health information, our results show that participants face difficulties in finding information on child development and education on websites (other than N&G) and web-based forums and navigating health and social care services when they have nonmedical care needs. In line with patient-centered care [84], future research may address several questions regarding child behavioral, developmental, and educational issues. To what extent can parents retrieve easy-to-read, listen to, and watch information in this field when needed? In what ways do websites and forums provide sufficient information to guide parents in managing these issues on their own when they desire and are able to do so? How do parents know who to ask for help when this information is insufficient? How can better understand what social care services are available and how they can be accessed?

Our results support approaches for improving information exchange between low-SES mothers and health or social care professionals, including (1) prescriptions of information that is considered trustworthy, easy to read, listen to, and watch; (2) information seeking by trusted relatives who have experience and higher level of eHealth literacy; and (3) referral to another professional, third party, when needed [84,85]. In other words, professionals can help low-SES mothers find, understand, evaluate, and use information because the provision of informational content alone does not usually satisfy the constraints of low education and empowerment among people of low SES [86].

Finally, the “Kate” narrative can be quickly read by decision policy makers, for example, web editors, and might encourage them to reach the underserved. In line with Reichwein et al [87], an ideal type can raise awareness and allow information providers, for example, web editors, to (1) tailor information content for the information needs and seeking behavior of specific target audience, such as low-SES mothers; (2) promote facilitators and overcome barriers to optimize information outcomes in the targeted low-SES audience; and (3) avoid stigmatization of people of low SES by openly reaching everybody (universalism).

Considering that narrative results of evaluations of interventions that show an impact on inequalities are especially useful for policy making [1], the “Kate” ideal type suggests the following main messages and policy recommendations. Web editors and experts can be encouraged to assess how valuable the informational content they share is from the perspective of low-SES people, providing thereby more equity-oriented content (easier-to-read, listen to, and watch information). In line with Luhmann theory of communication-based autopoietic self-referential interrelated social systems [88-91], the *Low Literacy Universal Precaution Principle* must be applied to enable trust and satisfactory communication (information exchanges) between Kate and a variety of social systems, such as education, health, and social services. People rely on website information content when they understand and trust it [92,93], whereas they do not use the content when they do not trust it, and often stop accessing it altogether [94]. In addition, O’Neill [95] specified that misplaced mistrust can be harmful. This applies to our work as follows: information providers and decision policy makers should promote trust in trustworthy information and should furnish resistance to mistrust in trustworthy information and trust in untrustworthy information.

In other words, information providers, such as web editors, must be encouraged, trained, and supported to provide trustworthy information using a plain language standard (readability grade 3-5), audio-assistance, and visuals [96]. This is important as a systematic review of 157 cross-sectional studies that assessed 7891 websites with consumer health information showed that the mean readability grade level ranged from grades 10 to 15 [96]. Thus, people of low SES and a low level of literacy are disproportionately disadvantaged regarding web-based information sources (readability grade superior to 5). Specifically, such inequalities remain with most governmental websites [97].

### Conclusions

This study suggests a main message, a feasible intervention, and policy recommendations that can be implemented across health care systems.

#### Message

Mixing quantitative and qualitative methods and results, this study shows that the perceived benefits from trustworthy easy-to-read, listen to, and watch web-based parenting information are higher for low SES mothers of young children compared with other mothers.

#### Intervention

Quantitative results are based on the IAM, a method that may be of interest to information providers. Specifically, assessing perceived outcomes of equity-oriented web-based information can help to verify that all people can obtain information they need, thus satisfying the democratic right to information [98-100].

#### Policies

This concurs to state that literacy-related education courses and continuing professional development activities are necessary across all health and social care disciplines. Our paper concludes with a call for applying universal plain language standards to all governmental and public websites, in an effort to reduce information-related inequalities and to humanize the development of communication technologies and the virtual world.

## Appendix

Multimedia Appendix 1 Example of the analysis process: from excerpts of interviews to themes.

Multimedia Appendix 2 Codebook: list of themes and subthemes (NVivo report).

## Abbreviations

IAM: Information Assessment Method
IP: internet protocol
N&G: Naître et grandir
SES: socioeconomic status

## References

[1] Farrer L, Marinetti C, Cavaco YK, Costongs C (2015). Advocacy for health equity: a synthesis review. Milbank Q.

[2] Baker DP, Leon J, Smith Greenaway EG, Collins J, Movit M (2011). The education effect on population health: a reassessment. Popul Dev Rev.

[3] Mikkonen J, Raphael D (2010). Social Determinants of Health: Canadian Perspectives.

[4] Mechanic D (2007). Population health: challenges for science and society. Milbank Q.

[5] Korenstein D, Falk R, Howell EA, Bishop T, Keyhani S (2012). Overuse of health care services in the United States: an understudied problem. Arch Intern Med.

[6] Suziedelyte A (2012). How does searching for health information on the internet affect individuals' demand for health care services?. Soc Sci Med.

[7] Smith S, Duman M (2009). The state of consumer health information: an overview. Health Info Libr J.

[8] Prescott J, Mackie L (2017). 'You sort of go down a rabbit hole...You're just going to keep on searching': a qualitative study of searching online for pregnancy-related information during pregnancy. J Med Internet Res.

[9] Erdem SA, Harrison-Walker LJ (2006). The role of the internet in physician–patient relationships: the issue of trust. Bus Horiz.

[10] Edwards M, Davies M, Edwards A (2009). What are the external influences on information exchange and shared decision-making in healthcare consultations: a meta-synthesis of the literature. Patient Educ Couns.

[11] Baker DW, Wolf MS, Feinglass J, Thompson JA, Gazmararian JA, Huang J (2007). Health literacy and mortality among elderly persons. Arch Intern Med.

[12] Amante DJ, Hogan TP, Pagoto SL, English TM, Lapane KL (2015). Access to care and use of the Internet to search for health information: results from the US national health interview survey. J Med Internet Res.

[13] Bessière K, Pressman S, Kiesler S, Kraut R (2010). Effects of internet use on health and depression: a longitudinal study. J Med Internet Res.

[14] Lauckner C, Hsieh G (2013). The Presentation of Health-related Search Results and Its Impact on Negative Emotional Outcomes. Proceedings of the SIGCHI Conference on Human Factors in Computing Systems.

[15] White RW, Horvitz E (2009). Cyberchondria. ACM Trans Inf Syst.

[16] Markoff J Microsoft Examines Causes of 'Cyberchondria'. The New York Times.

[17] McElroy E, Shevlin M (2014). The development and initial validation of the cyberchondria severity scale (CSS). J Anxiety Disord.

[18] El Sherif R, Pluye P, Thoër C, Rodriguez C (2018). Reducing negative outcomes of online consumer health information: qualitative interpretive study with clinicians, librarians, and consumers. J Med Internet Res.

[19] Case DO, O'Connor LG (2015). What's the use? Measuring the frequency of studies of information outcomes. J Assn Inf Sci Tec.

[20] Case D, Given L (2016). Looking for Information: a Survey of Research on Information Seeking, Needs, and Behavior. Fourth Edition.

[21] Robson A, Robinson L (2015). The information seeking and communication model. J Doc.

[22] Urquhart C, Turner J (2016). Reflections on the value and impact of library and information services. Perform Measure Metric.

[23] Glanz K, Rimer B, Viswanath K (2015). Health Behavior and Health Education. Fifth Edition.

[24] Richard C, Lussier M-T (2016). La communication professionnelle en santé.

[25] Thoër C, Lévy J (2012). Internet et santé.

[26] Connelly R, Speer M, Connelly RA, Turner T (2017). Health literacy and health communication. Health Literacy and Child Health Outcomes.

[27] Speer M, Connelly RA, Turner T (2017). Health literacy and child health outcomes: from prenatal to birth and infant stages. Health Literacy and Child Health Outcomes.

[28] Connelly R, Gupta A, Connelly RA, Turner T (2017). Health literacy universal precaution strategies for communication with all patients. Health Literacy and Child Health Outcomes.

[29] Gupta A, Speer M, Connelly RA, Turner T (2017). Health literacy effective health communication in pediatric practices health systems: creating shame-free environments patient-friendly institutions. Health Literacy and Child Health Outcomes.

[30] Turner T, Connelly RA, Turner T (2017). Health literacy medical education. Health Literacy and Child Health Outcomes.

[31] Ronson McNichol B, Rootman I, Raphael D (2016). Literacy and health literacy: new understandings about their impact on health. Social Determinants of Health: Canadian Perspectives.

[32] Weightman AL, Morgan HE, Shepherd MA, Kitcher H, Roberts C, Dunstan FD (2012). Social inequality and infant health in the UK: systematic review and meta-analyses. BMJ Open.

[33] Easton P, Entwistle VA, Williams B (2010). Health in the 'hidden population' of people with low literacy. A systematic review of the literature. BMC Public Health.

[34] Connelly R, Turner T (2017). Health literacy and child health outcomes. Champringer. ISBN.

[35] Baker S, Sanders MR, Morawska A (2016). Who uses online parenting support? A cross-sectional survey exploring Australian parents’ internet use for parenting. J Child Fam Stud.

[36] Jaks R, Baumann I, Juvalta S, Dratva J (2019). Parental digital health information seeking behavior in Switzerland: a cross-sectional study. BMC Public Health.

[37] Carolan M (2007). Health literacy and the information needs and dilemmas of first-time mothers over 35 years. J Clin Nurs.

[38] Lupton D (2016). The use and value of digital media for information about pregnancy and early motherhood: a focus group study. BMC Pregnancy Childbirth.

[39] Guendelman S, Broderick A, Mlo H, Gemmill A, Lindeman D (2017). Listening to communities: mixed-method study of the engagement of disadvantaged mothers and pregnant women with digital health technologies. J Med Internet Res.

[40] Dworkin J, Connell J, Doty J (2013). A literature review of parents’ online behavior. Cyberpsychology.

[41] Moon RY, Mathews A, Oden R, Carlin R (2019). Mothers' perceptions of the internet and social media as sources of parenting and health information: qualitative study. J Med Internet Res.

[42] Rothbaum F, Martland N, Jannsen JB (2008). Parents' reliance on the Web to find information about children and families: socio-economic differences in use, skills and satisfaction. J Appl Dev Psychol.

[43] Lloyd A, Anne Kennan M, Thompson KM, Qayyum A (2013). Connecting with new information landscapes: information literacy practices of refugees. J Doc.

[44] Knapp C, Madden V, Wang H, Sloyer P, Shenkman E (2011). Internet use and eHealth literacy of low-income parents whose children have special health care needs. J Med Internet Res.

[45] Hersberger JA (2013). Are the economically poor information poor? Does the digital divide affect the homeless and access to information?. Can J Inf Libr Sci.

[46] Chatman EA (1996). The impoverished life-world of outsiders. J Am Soc Inf Sci.

[47] Britz JJ (2016). To know or not to know: a moral reflection on information poverty. J Inf Sci.

[48] McCloud RF, Okechukwu CA, Sorensen G, Viswanath K (2016). Beyond access: barriers to internet health information seeking among the urban poor. J Am Med Inform Assoc.

[49] Palumbo R, Adinolfi P, Annarumma C, Musella M (2016). The special information needs of low health literate patients. Exploratory insights from an Italian survey. Proceedings Management in a Digital World.

[50] Rodrigo MJ, Muneton M. M, Suárez A (2016). Parental activities seeking online parenting support: is there a digital skill divide?. Revista de Cercetare si Interventie Sociala.

[51] Pluye P, El Sherif R, Granikov V, Hong QN, Vedel I, Galvao MC, Frati FE, Desroches S, Repchinsky C, Rihoux B, Légaré F, Burnand B, Bujold M, Grad R (2019). Health outcomes of online consumer health information: a systematic mixed studies review with framework synthesis. J Assoc Inf Sci Technol.

[52] Gregor S (2006). The nature of theory in information systems. MIS Q.

[53] Saracevic T (2007). Relevance: a review of the literature and a framework for thinking on the notion in information science. Part II: nature and manifestations of relevance. J Am Soc Inf Sci.

[54] Leonardi (2011). When flexible routines meet flexible technologies: affordance, constraint, and the imbrication of human and material agencies. MIS Q.

[55] Bush PL, Pluye P, Loignon C, Granikov V, Wright MT, Pelletier J, Bartlett-Esquilant G, Macaulay AC, Haggerty J, Parry S, Repchinsky C (2017). Organizational participatory research: a systematic mixed studies review exposing its extra benefits and the key factors associated with them. Implement Sci.

[56] Bush PL, Pluye P, Loignon C, Granikov V, Wright MT, Repchinsky C, Haggerty J, Bartlett G, Parry S, Pelletier J, Macaulay AC (2018). A systematic mixed studies review on Organizational Participatory Research: towards operational guidance. BMC Health Serv Res.

[57] Creswell J, Plano Clarke V (2018). Designing and conducting mixed methods research.

[58] Pluye P, Bengoechea EG, Granikov V, Kaur N, Tang DL (2018). A world of possibilities in mixed methods: review of the combinations of strategies used to integrate qualitative and quantitative phases, results and data. Int J Mult Res Approaches.

[59] Roelen K, Camfield L (2015). Mixed methods research in poverty and vulnerability: sharing ideas and learning lessons.

[60] Cappelleri J, Zou K, Bushmakin A (2014). Patient-reported Outcomes: Measurement, Implementation and Interpretation.

[61] Granikov V, Grad R, El Sherif R, Shulha M, Chaput G, Doray G, Lagarde F, Rochette A, Tang DL, Pluye P (2020). The Information Assessment Method: Over 15 years of research evaluating the value of health information. EFI.

[62] Pluye P, Granikov V, Bartlett G, Grad RM, Tang DL, Johnson-Lafleur J, Shulha M, Barbosa GMC, Ricarte IL, Stephenson R, Shohet L, Hutsul J, Repchinsky CA, Rosenberg E, Burnand B, Légaré F, Dunikowski L, Murray S, Boruff J, Frati F, Kloda L, Macaulay A, Lagarde F, Doray G (2014). Development and content validation of the information assessment method for patients and consumers. JMIR Res Protoc.

[63] Sherif RE, Roy P, Tang DL, Doray G, Dubois M, Bush P, Lagarde F, Pluye P (2017). The value of user feedback: Parent's comments to online health and well‐being information providers. Proc. Assoc. Info. Sci. Tech.

[64] Bujold M, El Sherif R, Bush PL, Johnson-Lafleur J, Doray G, Pluye P (2018). Ecological content validation of the Information Assessment Method for parents (IAM-parent): A mixed methods study. Eval Program Plann.

[65] Haynes SN, Richard DCS, Kubany ES (1995). Content validity in psychological assessment: A functional approach to concepts and methods. Psychological Assessment.

[66] Vogt DS, King DW, King LA (2004). Focus groups in psychological assessment: enhancing content validity by consulting members of the target population. Psychol Assess.

[67] Lowry R (2019). The confidence interval for the difference between two independent proportions web calculator.

[68] Newcombe RG (1998). Interval estimation for the difference between independent proportions: comparison of eleven methods. Stat Med.

[69] Tong A, Flemming K, McInnes E, Oliver S, Craig J (2012). Enhancing transparency in reporting the synthesis of qualitative research: ENTREQ. BMC Med Res Methodol.

[70] Bazeley P, Jackson K (2013). Qualitative data analysis with NVivo.

[71] Fereday J, Muir-Cochrane E (2016). Demonstrating Rigor Using Thematic Analysis: A Hybrid Approach of Inductive and Deductive Coding and Theme Development. International Journal of Qualitative Methods.

[72] Weber M (1995). Économie et société: les catégories de la sociologie.

[73] Pampalon R, Hamel D, Gamache P, Simpson A, Philibert MD (2014). Validation of a deprivation index for public health: a complex exercise illustrated by the Quebec index. Chronic Dis Inj Can.

[74] Manganello J, Gerstner G, Pergolino K, Graham Y, Falisi A, Strogatz D (2017). The Relationship of Health Literacy With Use of Digital Technology for Health Information: Implications for Public Health Practice. J Public Health Manag Pract.

[75] Keller J, McDade K (2000). Attitudes of low-income parents toward seeking help with parenting: implications for practice. Child Welfare.

[76] MacPhee D, Fritz J, Miller-Heyl J (1996). Ethnic Variations in Personal Social Networks and Parenting. Child Development.

[77] Henly JR, Danziger SK, Offer S (2005). The contribution of social support to the material well-being of low-income families. J Marriage and Family.

[78] Malone M, While A, Roberts J (2014). Parental health information seeking and re-exploration of the 'digital divide'. Prim Health Care Res Dev.

[79] Ziebland S, Wyke S (2012). Health and illness in a connected world: how might sharing experiences on the internet affect people's health?. Milbank Q.

[80] Kotchick B, Forehand R (2002). Putting parenting in perspective: a discussion of the contextual factors that shape parenting practices. J Child Fam Stud.

[81] Belsky J (1984). The determinants of parenting: a process model. Child Dev.

[82] Jones TL, Prinz RJ (2005). Potential roles of parental self-efficacy in parent and child adjustment: a review. Clin Psychol Rev.

[83] Montigny F, Lacharité C (2005). Perceived parental efficacy: concept analysis. J Adv Nurs.

[84] Gerteis M (1993). Through the patient's eyes: understanding and promoting patient-centered care.

[85] Niela-Vilén H, Axelin A, Salanterä S, Melender H (2014). Internet-based peer support for parents: a systematic integrative review. Int J Nurs Stud.

[86] Paige SR, Stellefson M, Krieger JL, Anderson-Lewis C, Cheong J, Stopka C (2018). Proposing a Transactional Model of eHealth Literacy: Concept Analysis. J Med Internet Res.

[87] Reichwein B, Wolmarans L, Nantayi L, Nassali F, Kakinda A, Musumba D, Nguyen TH, Baatsen P (2015). SegWeigh: a mixed-method approach to segmenting potential contraceptive user groups and meeting Family Planning 2020 goals. Int J Gynaecol Obstet.

[88] Luhmann N (1992). What is Communication?. Commun Theory.

[89] Luhmann N (1995). Social systems.

[90] Luhmann N (2006). La confiance: un mécanisme de réduction de la complexité sociale.

[91] Luhmann N (2001). La légitimation par la procédure.

[92] Vervier L, Valdez A, Ziefle M (2018). "Should I trust or should I go?" or what makes health-related websites appear trustworthy? - an empirical approach of perceived credibility of digital health information and the impact of user diversity. Proceedings of the 4th International Conference on Information and Communication Technologies for Ageing Well and e-Health.

[93] Chen X, Hay JL, Waters EA, Kiviniemi MT, Biddle C, Schofield E, Li Y, Kaphingst K, Orom H (2018). Health Literacy and Use and Trust in Health Information. J Health Commun.

[94] Dutton WH, Shepherd A (2006). Trust in the Internet as an experience technology. Information, Communication & Society.

[95] O'Neill O, Archard D, Deveaux M, Manson N N, Weinstock D (2013). Responses. Reading Onora O'Neill.

[96] Daraz L, Morrow AS, Ponce OJ, Farah W, Katabi A, Majzoub A, Seisa MO, Benkhadra R, Alsawas M, Larry P, Murad MH (2018). Readability of Online Health Information: A Meta-Narrative Systematic Review. Am J Med Qual.

[97] Scheerder A, van Deursen A, van Dijk J (2017). Determinants of Internet skills, uses and outcomes. A systematic review of the second- and third-level digital divide. Telematics and Informatics.

[98] Ford-Gilboe M, Wathen CN, Varcoe C, Herbert C, Jackson BE, Lavoie JG, Pauly BB, Perrin NA, Smye V, Wallace B, Wong ST, Browne For The Equip Research Program AJ (2018). How Equity-Oriented Health Care Affects Health: Key Mechanisms and Implications for Primary Health Care Practice and Policy. Milbank Q.

[99] Kutzin J (2013). Health financing for universal coverage and health system performance: concepts and implications for policy. Bull World Health Organ.

[100] Lehoux P, Roncarolo F, Silva HP, Boivin A, Denis J, Hébert R (2019). What Health System Challenges Should Responsible Innovation in Health Address? Insights From an International Scoping Review. Int J Health Policy Manag.

