# Supplementary material for: Outcomes of Equity-Oriented, Web-Based Parenting Information in Mothers of Low Socioeconomic Status Compared to Other Mothers: Participatory Mixed Methods Study
Source: J Med Internet Res. 2020 Nov 10;22(11):e22440. doi: 10.2196/22440 (PMC7685922; doi:10.2196/22440)
Supplement: Multimedia Appendix 1 [file jmir_v22i11e22440_app1.pdf]

APPENDIX 1

Example of the analysis process: From excerpts of interviews to themes and sub-themes

Pluye et al. Outcomes of equity-oriented online parenting information: a participatory mixed methods study comparing low SES with other mothers *Journal of Medical Internet Research (JMIR).*

Principal theme (level 1):
(B) Information on child development

Sub-theme (level 2):
Types of medias consulted by mothers

Sub-theme (level 3):

Web forums

Examples of excerpts of interviews coded to this sub-theme (content of the corresponding NVivo "node"):

[D01]: "There are groups on Facebook like… with discussions among mothers. I used to consult them often before but I don't anymore because I realized that it was stressing me. The mothers can be mean (laugh). I don't go on forums because… You can ask a question like 'Do you think it's normal that my 17-month-old daughter doesn't speak much?' And then half of people will tell you that it's perfectly normal, and the other half will say that it's stupid to even ask the question… So I decided that I don't go on web forums anymore. I unsubscribed from those things. In the end, it's not reliable […]"

[D01]: "But I don't do that anymore because I realized it's a loss of my time (laugh)."

[E02]: "There are lots of web forums too… There are mothers who say 'Yeah, this will happen' and 'I wonder about this or that concerning my daughter or son'. Plenty of people. There are always things that are repeated more often than others by people… So I'm saying: 'OK, I'll try that'."

[D12]: "Yes, I'll try Google first when I can… but then again, I don't want to ask a question on a forum if I can find the answer on Google myself. I hate when people do that (laugh). So I don't do that. But if I don't have a clue about what it is, then yes, I'll go on forums to ask my question."

Other level-3 sub-themes are websites, etc. (see list of themes and sub-themes in Appendix 2).
