# Supplementary material for: Outcomes of Equity-Oriented, Web-Based Parenting Information in Mothers of Low Socioeconomic Status Compared to Other Mothers: Participatory Mixed Methods Study
Source: J Med Internet Res. 2020 Nov 10;22(11):e22440. doi: 10.2196/22440 (PMC7685922; doi:10.2196/22440)
Supplement: Multimedia Appendix 2 [file jmir_v22i11e22440_app2.pdf]

APPENDIX 2

Codebook : List of themes and sub-themes (NVivo report)

Pluye et al. Outcomes of equity-oriented online parenting information: A participatory mixed methods study comparing low SES with other mothers *Journal of Medical Internet Research (JMIR).*

Note : Themes and subthemes have been translated from French to English for submitting this article.

Abbreviations :

- ADHD : Attention Deficit Disorder with or without hyperactivity
- ASD : Autism Spectrum Disorder
- IAM : Information Assessment Method
- N&G : Naitre et grandir

**Name**	**Number of interviews***	**Number of excerpts of interviews****
(A) Network and General Internet Use	*0*	*0*
Internet access	0	0
Internet cost	4	5
Difficult	1	1
Easy	34	37
Special needs of the child (or children)	16	16
Heart Problem	1	1
Development delay	1	1
Language delay	5	6
ADHD	5	7
ASD	2	2
Composition of the family	2	2
Intact two-parent	18	20
Recomposed two-parent	6	7
Age Group	0	0
more than 8 years old	7	7
0 to 12 months	3	4
>1 to 3 years	16	17
>3 to 5 years	9	11
>5 to 8 years	20	23
Single parent	14	16
Number of children	20	20
Personal use of the Internet	2	2
Beginning of Internet use	3	4
Per day	39	48
Per week	0	0
Consultation alone or accompanied	0	0
Accompanied	22	25
Alone	20	23

Preferred location for Internet consultation	1	1
Bedroom	4	4
Kitchen	0	0
Living room	6	6
Home	16	18
Educational setting	1	1
Public space	5	5
Workplace	4	4
Type of information consulted	3	3
Purchasing	8	13
News	6	7
Blogs	2	2
Entertainment	20	24
Child education	8	8
Studies	5	5
General Information	9	12
Itineraries-travel	2	2
Social Media	32	40
Recipes	8	9
Job Search	1	1
Health	8	9
Sites for children	2	2
Transportation	0	0
Timing of the consultation	0	0
Afternoon	3	3
During the day	3	4
Dinner Time	1	1
Morning	9	10
Anytime	8	9
Night	1	1
In a break	5	5
Evening	28	30
Type of device used	1	1
Cell	37	58
Desktop computer	17	19
Laptop computer	12	19
Tablet	14	20
(B) Information on Child Development	*0*	*0*
Access to resources	0	0
Difficult	12	14
Easy	18	26
Ease in using the media	0	0
No	0	0
Yes	8	8
Credibility of online information	36	54
Frequency of Information Needs since 1 year	3	3

more than 40 times	9	9
0 to 10 times	5	5
>10 to 40 times	3	3
Rarely	5	5
Very frequently (unspecified number)	13	14
Media and types of information	9	11
Information sharing	6	8
Process for searching information on the Internet	7	8
Search in mother tongue first	10	10
Search in English first	3	4
Comparison of different sources	12	17
Googling	20	30
Following a list of sources	5	5
Reason for Information Needs	5	6
Activities for children	7	8
Child development	15	23
Child education	7	8
Child health	15	17
Finding resources	3	3
Verifying or validating own experience	5	5
Parental life	6	6
Social support network	0	0
Friends	4	4
Parents	9	13
Other relatives	2	2
Neighbors	1	1
Colleagues at work	0	0
Others	4	6
Quality of the social network	1	1
Poor	6	6
Rich	2	3
Favourite sites on child development	18	22
Sources consulted for information purpose	5	5
Friends	26	36
Parents	29	42
Grandma	3	3
Colleagues	1	1
Educator	11	15
Teacher	6	7
Internet	31	57
Book Living Better with Your Child	2	2
Community organization	3	7
Health and social services (professionals)	5	5
Local Community Center	4	5
Dentist	0	0

Pediatric Hospital	1	1
Nurse	4	5
811 Health/Social Infoline 24/7	21	28
Physician	23	33
Orthopedagogue	4	4
Speech-Language Pathologist	5	6
Pharmacist	15	17
Psychoeducator	3	4
Psychologist or therapist	3	3
Social worker	6	9
Emergency Room	5	8
Special education technician	1	1
Types of media consulted	4	5
Web Forums	3	3
Websites	28	37
Books	6	8
Specialized magazines	32	38
Monographs	5	6
Radio	26	27
Social networks	7	9
TV	37	39
(C) N&G	*0*	*0*
Recommended addition of visuals	0	0
No	10	13
Yes	0	0
Drawings	4	4
Cultural diversity	1	1
Forum	2	2
Photos	13	14
Videos	30	40
Application of the N&G information	0	0
No	1	1
Yes	12	17
Cultural diversity	0	0
Photos	0	0
Videos	1	1
Appreciation of the N&G information	2	2
Non-normative aspect	4	5
Normative aspect	1	1
Proposed solutions	8	8
Validation of own experience	3	4
Audio Player appreciation	5	5
Never noticed it on the page	10	10
No	14	17
Yes	21	25
General appreciation of the N&G website	0	0

Negative aspects	6	6
Positive aspects	30	52
Clarity of information on the N&G webpages	0	0
Ambiguous	1	1
Clear	33	44
Understandability of the N&G information on the pre-identified page	29	39
Knowledge of the N&G magazine	2	2
No	0	0
Yes	8	8
Knowledge of the N&G website	0	0
No	10	12
Yes	9	11
Credibility of the N&G information	1	1
Credible	27	30
Not very credible	0	0
Availability of information elsewhere	1	1
No	5	5
Yes	21	22
More or less	6	6
Potential places to consult N&G	3	3
Other location	18	20
Local public library	36	41
Local community center	35	40
Child care center	4	4
Home	6	6
Hospital	2	2
Place of worship	20	20
Community organization	30	38
Pediatrician	2	3
Pharmacy	35	38
Workplace	1	1
Completeness of information on the pre-identified page	2	2
Complete	25	33
Incomplete	13	17
Ease of use N&G	0	0
No	4	4
Yes	33	51
Form of presentation of N&G information	2	3
Comic strip	7	9
Photonovel	2	3
Status quo	2	2
Video	10	10
Frequency of N&G use	14	15
Printing N&G information	0	0

No	25	27
Yes	5	6
Screenshot	20	21
N&G Newsletter	3	5
N&G characteristics to be maintained	5	6
Colors	3	4
Ease of use	11	14
Search engine	3	3
Tabs	22	23
Photos	4	4
Question(s) to keep	0	0
Section A to Z	2	2
Subtitles and sections	7	7
N&G characteristics to be modified	11	13
Age Categories	2	3
Chat room	2	2
Content	3	3
Forum (to be added)	4	6
Character size	4	4
Logos	1	1
Home page	5	5
Portrait or family example	1	1
Rehearsals	1	1
Section from A to Z	1	1
Resources section	1	1
Video	2	2
Sharing N&G pages with others	2	2
No	2	2
Yes	21	27
Reasons for consulting N&G	3	3
Child behaviour	1	1
Pregnancy	2	2
Newsletter	1	1
N&G Magazine	1	1
Reasons for not consulting N&G	1	2
Uncomfortable on a cell phone	0	0
Lack of time	0	0
Lack of interest	0	0
Agreement with N&G recommendation	1	1
No (justifications)	0	0
Yes (justifications)	25	25
Maybe	1	1
Attractivity of the N&G website	0	0
No	0	0
Colors	5	7
Logo	1	1

Too many headings on the homepage	1	1
Yes	11	12
Colors	7	10
Logo	1	1
Left side menu	2	2
Tabs	0	0
Tabs by age groups	15	15
Photos	4	4
Sections A to Z	1	1
Sleek style	3	4
Suggestions for improvement	13	25
Type of device used to consult N&G	0	0
Cell	3	3
Desktop computer	1	1
Laptop computer	0	0
Tablet	0	0
Usefulness of the information from N&G	2	2
Examples of how the information is used in the participant's life	15	19
No	5	7
Yes	28	37
(D) IAM	*0*	*0*
Suggested improvement for IAM	3	6
Add answer choices	2	2
Add a "Follow-up" section	1	1
Configure cell phones to access the IAM	1	1
Colors	10	13
Move the tab	4	4
Interactivity	10	12
Number of questions	4	6
Tab	15	20
Reduce the number of answer choices	6	9
Replace questions with a rating	0	0
Sub-questions	8	9
Use stars to rate the answers	4	4
General appreciation IAM	17	21
Understanding the IAM	1	1
No	3	3
Yes	24	25
Alternative form of presentation of the IAM	1	1
Undesirable	16	18
Desirable	8	8
Behaviour regarding Internet evaluation forms	4	4
Usually completed	4	4
Usually not completed	6	6
Reading the IAM	1	1

Difficult	5	5
Easy	22	23
Location of the IAM tab	2	2
Adequate	9	11
Inadequate	27	33
The tab does not appear	4	4
Length of IAM	0	0
Adequate	32	34
Inadequate	5	6
IAM characteristics to be maintained	20	25
IAM characteristics to be modified	26	39
IAM Audio Support	0	0
Undesirable	7	7
Desirable	9	9
IAM visual support	22	26
Type of device	0	0
Cell	1	1
Desktop computer	0	0
Laptop computer	0	0
Tablet	0	0
(E) Socio-demographic information	*0*	*0*
Socio-demographic information	24	31

Legend
*Number of interviews with at least one excerpt assigned to the corresponding theme or sub-theme.
**Total number of excerpts of interviews assigned to the corresponding theme or sub-theme.
